# Fatigue Performance of Metal–Composite Friction Spot Joints

**DOI:** 10.3390/ma14164516

**Published:** 2021-08-11

**Authors:** Seyed Mohammad Goushegir, Jorge F. dos Santos, Sergio T. Amancio-Filho

**Affiliations:** 1 Solid State Materials Processing, Institute of Materials Mechanics, Helmholtz-Zentrum Hereon, Max-Planck-Str. 1, 21502 Geesthacht, Germany; mgoushegir@gmail.com (S.M.G.); jorge.dos.santos@hereon.de (J.F.d.S.); 2 BMK Endowed Professorship for Aviation, Institute of Materials Science, Joining and Forming, Graz University of Technology (TU Graz), 8010 Graz, Austria

**Keywords:** friction spot joining, composite materials, aluminum and alloys, surface preparation, mechanical properties, fatigue life

## Abstract

Friction spot joining is an alternative technique for joining metals with polymers and composites. This study investigated the fatigue performance of aluminum alloy 2024/carbon-fiber-reinforced poly(phenylene sulfide) joints that were produced with friction spot joining. The surface of the aluminum was pre-treated using various surface treatment methods. The joined specimens were tested under dynamic loading using a load ratio of R = 0.1 and a frequency of 5 Hz. The tests were performed at different percentages of the lap shear strength of the joint. Three models—exponential, power law, and wear-out—were used to statistically analyze the fatigue life of the joints and to draw the stress–life (S–N) curves. The joints showed an infinite life of 25–35% of their quasi-static strength at 10^6^ cycles. The joints surpassing 10^6^ cycles were subsequently tested under quasi-static loading, showing no considerable reduction compared to their initial lap shear strength.

## 1. Introduction

Joining lightweight metal alloys with glass-fiber- and carbon-fiber-reinforced polymer (GFRP and CFRP) composites has attracted the attention of researchers and relevant industries, such as transportation, in recent years. The obvious reason is the ability to reduce the weight of a structure by using lightweight yet strong materials. Several methods that are suitable for joining metals to composites are available, such as mechanical fastening and adhesive bonding. Because of some limitations of these conventional techniques, such as a weight penalty, inhomogeneous stress distribution, and environmental-related issues (e.g., generation of vaporized chemical compounds during processing and application of adhesives), several alternative joining technologies were developed and introduced in the last decade. These new processes, such as laser joining [1], ultrasonic welding [2], and induction welding [3], use different energy sources to weld a metal part to a thermoplastic polymer. In these methods, the metal part is heated below its melting point. The generated heat conducts to the thermoplastic part, melting a volume of the polymer, which after a cooling phase and consolidation, a joint is formed. These processes were categorized as welding-based joining technologies (WB-JT) because of the melting and reconsolidation of the thermoplastic part [4]. A new class of WB-JT, namely, friction-based joining, has grown in importance for the international manufacturing and scientific communities due to the high energy efficiency, resultant mechanical performance, and short joining times [5].

Friction spot joining (FSpJ) is one of the welding-based metal–composite joining techniques that were developed and patented by Helmholtz-Zentrum Hereon in Germany [6]. FSpJ presents several advantages, including fast joining cycles, using simple and available equipment, the possibility of automation, and being an environmentally friendly process [7]. In the previous investigations, different aspects of the joints were described. The general description of the process, feasibility studies, and microstructural features of the friction spot joints were discussed [8,9,10]. The process was optimized for different combinations of materials, and the influence of the process parameters on the weld formation and mechanical performance was addressed [11,12,13]. Furthermore, investigations on the influence of various metal and composite surface pre-treatments on the quasi-static strength of the joints [14,15], the interfacial bonding mechanisms using X-ray photoelectron spectroscopy (XPS) [16], and the use of film interlayers [17], e.g., to improve stress distribution at the metal–composite interface, were carried out. The influence of engineering corrosion in FSpJ is an extensively reported topic [18]. Furthermore, detailed fracture micro-mechanisms of the metal–composite hybrid joints were studied and experimentally described [19] and computationally modeled [20]. Impact resistance [21] was additionally investigated and described for single-lap specimens. However, all these investigations were conducted under impact or quasi-static loading, and information regarding the fatigue properties of the metal–composite friction spot joints remains a knowledge gap.

Moreover, to the best knowledge of the authors, the only published data regarding the fatigue behavior of the welding-based joining techniques were reported by Balle et al. [22] for the ultrasonic welding of aluminum alloy 5754 and carbon-fiber-reinforced polyamide (CF-PA66). The aluminum alloy was acid pickled prior to the welding. The S–N (stress–life) curve of the joint was presented, although the employed stress ratio (R = minimum cyclic stress/maximum cyclic stress) was not accurately given. The authors used a power-law model to fit the fatigue data and concluded that the fatigue limit was approximately 35% of the respective quasi-static strength for the selected pre-treatment. Therefore, there is still much to learn about different aspects of the welding-based joining techniques, including their mechanical performance under cyclic loading.

There are primarily two approaches that are used to analyze the fatigue life of a joint [23]: first, via the life evaluation and S–N curve analysis, and second, via the analysis of fatigue crack growth. Various models have been proposed to analyze and predict the fatigue strength (or life) of a material or a joint. One of the very first models developed to fit the S–N curve of metallic structures was introduced by Basquin [24]. This was essentially a power-law model (in the form of σ = bN^−a^, where σ is the fatigue strength, N is the respective fatigue life, and a and b are model parameters), which is used for fatigue data obtained under uniaxial loading conditions [25]. However, it was stated that this model could fit the data for high cycle fatigue (HCF) but does not properly fit the data when a low number of cycles is used [26]. Therefore, the model was improved via the addition of new parameters to better control the shape of the S–N curve in both the low- and high-cycle fatigue regions [27]. As a first attempt, the Basquin model was used to analyze the fatigue life behavior of composites. However, due to the more complex damage mechanisms of composites under fatigue loading, modified models were derived, for example, by Epaarachchi and Clausen [28].

Another issue with the fatigue analysis of composite materials was the high standard deviation that is associated with fatigue life [25,27]. New models were therefore proposed that considered the probabilistic nature of the fatigue life of composite materials [29,30,31]. Of particular importance are the differences between assumptions in each model. In a model established by ASTM [29], it is assumed that fatigue life follows a normal distribution at each stress level, whereas in the other models [30,31], a Weibull distribution is assumed.

Apart from the different models that are used to obtain a reliable S–N curve for the fatigue life analysis of a material or joint, various aspects, such as joint design [32,33,34] and joining partners [23], may influence the fatigue strength of a joint. Surface treatments are also among the influential parameters that affect the fatigue behavior of a joint. Several studies have shown that chemical pre-treatments can improve the fatigue life of adhesively bonded metal–metal [35] and metal–polymer [23,36] joints. Improved adhesion at the interface was reported [23,36] to positively influence not only the quasi-static strength of the joints but also their fatigue life. Furthermore, Bland et al. [37] investigated the influence of pre-treatments on the wet fatigue behavior of adhesively bonded aluminum joints. Although in wet conditions (a harsher environment), their findings pointed out that a phosphoric acid anodizing (PAA) pre-treatment with a subsequent primer application had the best fatigue performance followed by stand-alone PAA pre-treatment and sandblasting.

This work investigated the fatigue performance of single-lap shear (SLS) friction spot joints based on the S–N curve life analysis under constant amplitude loading. Three fitting models were used to obtain the S–N curves of the tested samples. The fracture surface of the joints after fatigue testing was analyzed using scanning electron microscopy (SEM).

## 2. Materials and Methods

### 2.1. Materials

Two-millimeter thick sheets of aluminum alloy 2024-T3 (AA2024-T3) that were supplied by Constellium, France, with the nominal chemical composition (wt%) 0.1 Si, 0.17 Fe, 4.55 Cu, 0.45 Mn, 1.49 Mg, <0.01 Cr, 0.16 Zn, 0.021 Ti, and Al balance was used as the metal component. The alloy exhibits a high strength-to-weight ratio, good fatigue resistance and damage tolerance, high fracture toughness, and good formability [38]. This alloy is mainly used in the primary structures of aircraft.

The selected composite part was a carbon-fiber-reinforced poly(phenylene sulfide) (CF-PPS) containing 50 vol% of carbon fibers. This was a laminated composite supplied by TenCate, the Netherlands, consisting of 5 harness-woven quasi-isotropic laminates. The composite consisted of 7 plies of carbon fibers in the following sequence: [(0,90)/(±45)]3/(0,90) corresponding to a nominal thickness of 2.17 mm. CF-PPS is considered a high-performance semi-crystalline thermoplastic composite that is mainly used in primary and secondary aircraft parts because of its high strength, rigidity, chemical resistance, and low water absorption [39,40,41]. AA2024-T3 and CF-PPS were selected because of their applications in the aircraft industry.

### 2.2. Methods

#### 2.2.1. Friction Spot Joining (FSpJ)

Friction spot joining was used to join the aluminum with the composite using displacement-controlled equipment (RPS100, Harms&Wende, Hamburg, Germany). The principles of the process were previously discussed thoroughly [7,9,10,11,17,42]. In brief, a non-consumable rotating tool was plunged into the aluminum (the upper sheet), generating frictional heat and plasticizing a volume of the aluminum around the tool. The plasticized aluminum formed a metallic nub in the form of an undercut at the interface with the composite as a result of the axial movement of the tool. The metallic nub generated macro-mechanical interlocking between the joining parts, which is known as one of the bonding mechanisms in FSpJ specimens. At the same time, the frictional heat was conducted from the aluminum to the interface with the composite. This led to the melting of a layer of the CFRP matrix, which after consolidation during the cooling phase, generated adhesion forces between the aluminum and composite. Such adhesion forces also acted as a bonding mechanism.

**Figure 1:**
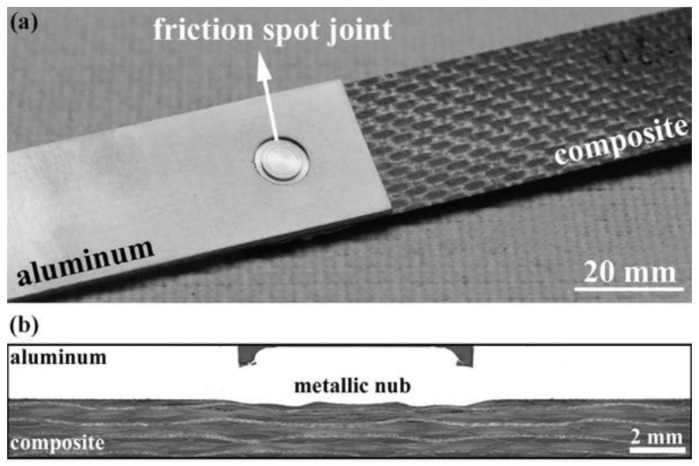
(**a**) Top view of a sound FSp join after consolidation and (**b**) a typical cross-section of an aluminum–composite FSp joint, indicating the metallic nub.

Figure 1 shows an aluminum–composite friction spot joint, as well as the metallic nub in the cross-section of the joint at the center of the spot.

An overlap configuration was used to produce the joints. Both aluminum and CF-PPS were cut in 100 mm × 25 mm specimens, and an overlap length of 25 mm was selected. Four aluminum surface pre-treatments were selected to carry out the fatigue experiments; these were sandblasting (SB), sandblasting with subsequent conversion coating (SB + CC), phosphoric acid anodizing (PAA), and PAA with subsequent application of the primer (PAA-P). The treatments were applied on the surface of the aluminum before the joining process to understand the influence of the surface pre-treatment on the fatigue performance of the joints. For more information about the detailed procedures used to treat the surface of the specimens, refer to [14]. In the case of the CF-PPS, the specimens were cleaned by wiping the surface with acetone.

An optimized set of joining parameters was selected to produce the single-lap shear AA2024-T3/CF-PPS specimens based on a previous investigation [12]. The selected parameters were the rotational speed of the tool: 2900 rpm, plunge depth into the aluminum sheet: 0.8 mm, joining time: 4 s, and joining pressure: 0.3 MPa.

#### 2.2.2. Mechanical Testing

**Figure 2:**
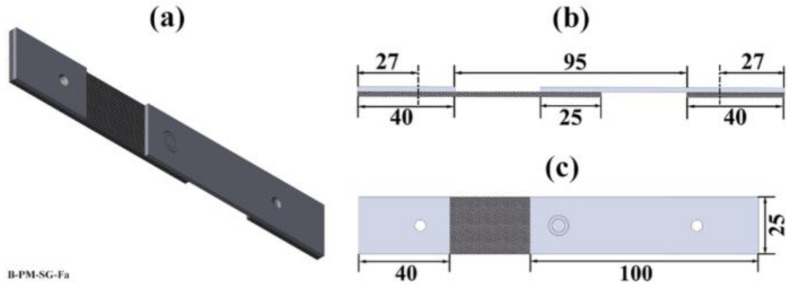
(**a**) Schematic illustration of the geometry of the fatigue specimen; the respective dimensions used in this work from (**b**) the side view and (**c**) the top view. All dimensions are in millimeters. Reprinted from [7] with permission; Copyright Springer Nature 2021.

A single-lap shear geometry was used to investigate the mechanical properties of the joints under fatigue loading. Aluminum and composite specimens were machined with dimensions of 100 mm × 25 mm (Figure 2). Two end tabs (each 40 mm × 25 mm) were used to align the specimens during the testing. Therefore, a free length of 95 mm was obtained to perform the fatigue experiments. The specimens were fixed in the testing machine using two rivets at a distance of 27 mm from each edge. Load-controlled servo-hydraulic equipment with a load capacity of 25 kN was employed to perform the experiments. Constant amplitude fatigue testing at a frequency of 5 Hz and a tension–tension load ratio of R = 0.1 was used in this work. Various load levels (in accordance with the quasi-static strength of the joints) were chosen to obtain the fatigue life of the joints in the range of 10^3^ to 10^6^ cycles. At least three samples were tested for each load level, and the experiments were conducted at room temperature.

The joints that survived one million fatigue cycles without failure (called run-out specimens) were subsequently tested under quasi-static conditions. The results are reported as the residual strength of the joints after one million fatigue cycles.

For the quasi-static testing, a universal testing machine (Zwick Roell model 1478) with a load capacity of 100 kN was used. The traverse test speed was 1.27 mm/min, and the tests were performed at room temperature according to ASTM D3163-01. The average ultimate lap shear force (ULSF) of the joints was obtained as the peak force that a joint can bear before final failure.

#### 2.2.3. Microscopy

To analyze the fracture surface of the joints after fatigue failure, SEM (Quanta^TM^ FEG 650 equipment, FEI, Hillsboro, OR, USA) was used. For that purpose, a voltage of 5 kV, a spot size of 3, and a working distance of 15 mm were set.

### 2.3. Description of the Fatigue Fitting Models

In this work, three fitting procedures were chosen to obtain S–N curves; first, the exponential model, also known as the semi-log or Lin-Log; second, the power-law model, also referred to as the Log-Log model in the literature; and finally, the wear-out model based on the Sendeckyj approach. These models are briefly described in this section.

#### 2.3.1. Exponential and Power-Law Models

Exponential and power-law models are the most widely used methods to analyze the fatigue life of overlap joints [22,43,44]. ASTM standard practice E739-10 [29] thoroughly explains the procedure of fitting the fatigue data using the mentioned models. The following equations are used to define the shape of the S–N curve based on the above ASTM standard:

(1) $$\log\;\left(N\right)\;=\;a\;+\;\mathrm{bF}$$

(2) $$\log\;\left(N\right)\;=\;c\;+\;\mathrm{dlog}\left(F\right)$$

where N is the fatigue life; F refers to the applied force at a constant amplitude; and a, b, c, and d are the model parameters determined using a linear regression model. Equation (1) is employed to fit the exponential model, whereas Equation (2) is used for the power-law model. A very important consideration for estimating model parameters is that the fatigue life is the dependent parameter, whereas force is an independent parameter. Moreover, in these models, a linear relationship between the applied force and the obtained fatigue life is assumed [29]. In addition to the S–N linear relationship, it is assumed that the logarithm of the fatigue life (N) follows a normal distribution [25,27,29]. Finally, it is recommended that no run-outs are used to estimate the S–N curve. Run-outs are samples that survive a pre-defined number of cycles and testing is stopped before their failure.

#### 2.3.2. Wear-Out Model

Although the exponential and power-law models were widely used to fit fatigue data, they do not consider the probabilistic nature of the fatigue properties of composite materials. It is reported that the fatigue lives of composite materials display high levels of scattering, which forced the development of more complex models to fit the fatigue data and thus obtain statistically reliable S–N curves [27]. Wear-out or strength degradation models were proposed with various approaches to derive S–N curves, taking into account the probability of failure under fatigue loading.

Sendeckyj proposed a wear-out model that has a couple of advantages for composite materials [25,27,30]. The first advantage, as already mentioned, is the consideration of the probabilistic behavior of composite materials under fatigue loading. The second is that run-out specimens can also be used in the analysis to determine the S–N curve.

In the model proposed by Sendeckyj, the fatigue strength data are converted to equivalent static strength (ESS) using Equation (3) [25,27,30]:

(3) Fe = FmaxFrFmax1S′ + N − 1CS′

where F_e_ is the equivalent static force, F_max_ refers to the maximum applied force, F_r_ is the residual strength of the run-out specimens, N is the fatigue life of each specimen at the applied stress, and C and S′ are the model parameters. C and S′ control the shape of the S–N curve in the low-cycle fatigue regime and the slope of the curve, respectively. Note that in this work, the equivalent static force was calculated. However, the term ESS is used due to its convention in the literature.

In the wear-out model, in contrast to exponential and power-law models, it is assumed that the ESS data follow a two-parameter Weibull distribution. Therefore, a two-parameter Weibull distribution is fitted to the ESS data to obtain the Weibull shape parameter (α) and scale parameter (β). An iterative process needs to be performed based on different values of C and S′ to obtain the maximum shape parameter of the Weibull distribution (α_f_). Finally, the S–N curve can be obtained from Equation (4) (which has the form of a power equation) for the desired reliability level [30]:

(4) Fmax = β−lnRN1αfNf − AC−S′

where α_f_ and β refer to the maximum shape and scale parameters, respectively, of the fitted Weibull distribution; R(N) is the selected reliability value; N_f_ is the fatigue life; and A is calculated as follows [30]:

(5) $$A\;=\;-\frac{1\;-\;C}{C}$$

A major assumption in this model is that the failure mode of the fatigue specimens should be similar to quasi-static samples, and no competing failure modes are occurring.

For more information on fitting procedures using the above-mentioned models, refer to [29] for the exponential and power-law models and to [30] for the wear-out model.

## 3. Results and Discussion

Before explaining the results of the fatigue life evaluation of the joints, it is worth mentioning that the quasi-static strengths of the specimens using various surface pre-treatments were evaluated [14]. The results showed that PAA-P specimens yielded the highest lap shear strength with an average ULSF of 8788 ± 62 N [14]. For SB, SB + CC, and PAA specimens, the average ULSFs obtained were 2324 ± 151 N, 3276 ± 352 N, and 3276 ± 115 N, respectively [14]. Different load levels (25–75%) as a function of the ULSF of each surface-pre-treated specimen were selected to perform fatigue experiments.

### 3.1. Fatigue Life Analysis of the Friction Spot Joints

This section describes the results obtained from the fatigue experiments of the friction spot joints using four selected surface pre-treatments and the life analysis based on the selected models. It is worth mentioning that all the results in this chapter are presented as load–life (F–N) graphs. However, the term S–N curve (strength–life) is used due to its widespread usage in the literature.

**Figure 3:**
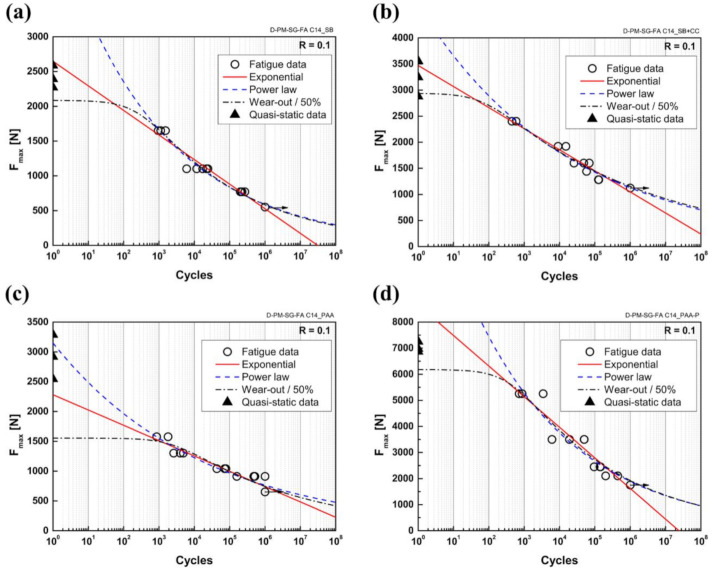
Derived S–N curves based on exponential, power-law, and wear-out models for the (**a**) SB, (**b**) SB + CC, (**c**) PAA, and (**d**) PAA-P specimens.

The S–N curves derived using the examined models for the selected surface pre-treatments are illustrated in Figure 3. The detailed fatigue data from the S–N curves discussed in this manuscript are presented in Appendix A and Appendix B. In addition to the fatigue data (indicated by the open circles) and the derived S–N curves, the initial quasi-static strength of three replicates for each joint (indicated by solid triangles) was also plotted for comparison. Note that for the wear-out model, 50% reliability was used, which is usual for composite materials [27].

A major assumption in the wear-out model is that the failure mode of the fatigue specimens should be similar to the quasi-static samples and that no competing failure modes are presented [25]. This assumption is valid in the case of the friction spot joints, as was observed from the fracture surface of the joints, which is discussed later when considering the fatigue failure behavior.

In all cases, the behavior of the three models was similar across the range of the experiments (10^3^–10^6^ cycles) and all the models were capable of fitting the fatigue data. One can also observe that the wear-out model exhibited a similar trend to the power law, except in the low-cycle fatigue (LCF) range. This was expected because the wear-out model also obeys a power law, except that parameter C controls the shape of the curve in the LCF range, leading to a deviation from the power-law model. In the range of high-cycle fatigue (HCF) above 10^6^ cycles, the exponential model seemed to be a very conservative approach because it predicted a limited fatigue life under load zero. The same behavior for the exponential model was reported by Khabbaz for adhesively bonded CFRP joints [27]. The power law and the wear-out models tended to follow the experimental data in this range. However, in the LCF regime below 10^3^ cycles for the SB and SB + CC specimens, the exponential model could more appropriately predict the fatigue life, whereas, at one cycle, the predicted results were in the range of the quasi-static strength of the joints. Nevertheless, the comparison between the fatigue strength (at one cycle) and the quasi-static strength should be treated with care since the strain rate during both testings could be different and may influence the obtained strength [25]. The wear-out model gave the lowest predicted strength for LCF, followed by the exponential and power-law models in all surface-pre-treated joints. The power-law model predicted very high strength for the SB, SB + CC, and PAA-P specimens (even higher than their respective quasi-static strength), whereas it could still predict the strength appropriately for the PAA specimens. Finally, the wear-out model predicted the strength of the joints in the LCF regime close to the quasi-static strength for the SB, SB + CC, and PAA-P samples, but it appeared to be very conservative for the PAA specimens.

### 3.2. Influence of Surface Pre-Treatment

**Figure 4:**
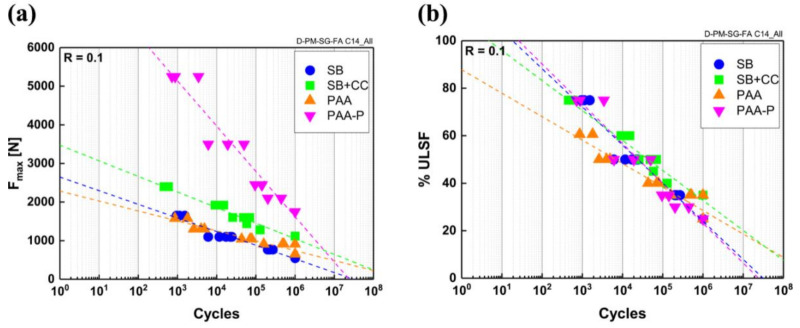
(**a**) S–N curves of the various surface-pre-treatment specimens based on the exponential model. Reprinted from [7] with permission; Copyright Springer Nature 2021. (**b**) S–N curves of the specimens as a percentage of their initial quasi-static strength (ultimate lap shear force).

As all the models showed an effective fitting in the experimental range and since the exponential model has frequently been used in the literature, both for metallic and composite structures, it was selected to compare the fatigue behavior of the pre-treated specimens. The comparison is shown in Figure 4a. The friction spot joints that were produced using the PAA-P surface pre-treatment exhibited the highest fatigue strength in the range of experiments. SB, SB + CC, and PAA specimens displayed similar behaviors. However, SB + CC joints showed slightly better performance compared to the stand-alone SB and PAA samples in the entire range of analysis. Therefore, the fatigue strength of the joints followed a similar trend to the quasi-static strength of the friction spot joints, as reported earlier and in detail in [14].

It is obvious from Figure 4a that the PAA-P specimens had a much higher fatigue strength compared to the other surface pre-treatments in the LCF regime (10^3^ cycles), showing very similar performance to the pre-treated joints under quasi-static loading. However, the fatigue strength of the PAA-P samples tended to approach the rest of the surface pre-treatments in the HCF range (10^6^ cycles). The reason for such a steep decrease in the fatigue strength of the PAA-P joints is believed to be related to the secondary bending effect that is present in the SLS joint geometry [43,45]. It has been reported that out-of-plane peel stresses form at the edges of an SLS joint during each cycle of fatigue testing [43,45]. The generation of peel stresses reduces the fatigue performance of the SLS joints and leads to premature failure. Furthermore, it has frequently been discussed in the literature that shear stresses are maximized at the edges of an SLS joint under shear loading, whereas these stresses tend toward zero at the center of the joint. The combination of peak shear stresses and peel stresses at the edges of the joint may drastically reduce the performance of an SLS joint under cyclic loading. It is known that the externally applied loads directly influence these induced stresses through the bending moment [46,47,48,49]. Since the PAA-P specimens showed a much higher quasi-static strength compared to the other surface pre-treated specimens in this work, the corresponding externally applied fatigue loads were also higher. This led to the formation of much higher peak shear and peel stresses at the edge of the friction spot joint, particularly in the HCF regime, resulting in a steeper slope of the S–N curve for the PAA-P joints.

Furthermore, the fatigue performance of the PAA specimens was slightly better than the SB specimens in the HCF range. This may be attributed to the influence of surface roughness on stress concentration and fatigue life reduction. It was reported by Shahzad et al. [50] that high surface roughness reduced the fatigue life of the aluminum alloy 7010 single material, especially in the HCF regime. With FSpJ, it is believed that the sharp asperities and ridges on the aluminum surface that are due to the SB pre-treatment acted as stress concentration points. This, in turn, facilitated crack nucleation, leading to the reduction of fatigue life, particularly in the HCF regime. However, for the PAA specimens, due to the lower surface roughness, the slope of the S–N curve was less steep. Moreover, SB generates very sharp-edged asperities compared to the more smooth oxide layer that is produced by the PAA pre-treatment. Such sharp edges may also facilitate stress concentration and crack nucleation. The same trend as for the SB specimens can be seen for the SB + CC specimens, but the slightly higher fatigue strength in the HCF range compared with the PAA specimens could be related to the presence of chemical bonding in addition to mechanical interlocking [14,16]. Nevertheless, and similar to the above discussions, generated peel and maximum shear stresses were smaller for SB, SB + CC, and PAA specimens when compared with the PAA-P samples, leading to less steep S–N curves.

Table 1 shows the fatigue strength of the friction spot joints using different pre-treatments at 10^5^ cycles of fatigue life. The aircraft industry usually uses 10^5^ cycles as a reference to compare the fatigue performance of different designs. The results show that the models predicted quite similar strengths for each surface pre-treatment. Furthermore, the PAA-P specimens gave the highest fatigue strength as a result of the very strong carbon–carbon chemical bonds that were formed at the interface, as reported in [14]. The SB + CC specimens ranked second in fatigue performance, followed by the PAA and SB specimens.

Additionally, comparing the fatigue life of the joints as a function of their initial quasi-static strength (Figure 4b), PAA-P and SB specimens showed steeper curves compared to the PAA and SB + CC specimens. This was due to the explanations mentioned above of the generated peel stresses (PAA-P samples) and stress concentration (SB samples) as a result of the sharp-edged asperities. At 10^5^ cycles (the range of application for designing primary aircraft structures), all surface pre-treatments showed a fatigue life that was approximately 40% of their initial quasi-static strength. The indefinite fatigue life of the joints at 10^6^ cycles was obtained at about 25% of the initial lap shear strength for the SB, PAA, and PAA-P specimens and 35% for the SB + CC samples. For the SB + CC specimens, one expects lower peel stresses and chemical bonding because of the conversion coating. Moreover, applying conversion coating after SB might smoothen the sharp edges of the asperities, leading to a reduction in the local stress concentration compared to the stand-alone SB samples.

### 3.3. Residual Strength

**Figure 5:**
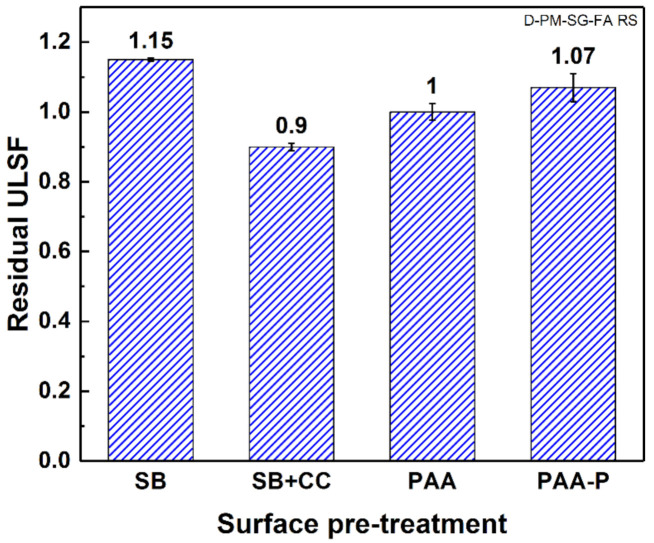
Residual strength of the joints for the surface pre-treatments after 10^6^ cycles of fatigue loading.

To obtain the residual strength of the joints surviving one million cycles of fatigue, the specimens were subsequently tested under quasi-static lap shear testing. As can be observed in Figure 5, the residual strength of the fatigued specimens did not show any significant reduction. Even for the SB and PAA-P joints, the residual strength was slightly higher compared to the initial strength of the joints. However, considering the standard deviation of the experiments, in most cases, neither the increases nor the decreases in residual strength were significant. As damage accumulation during fatigue testing often reduces the residual strength of a joint [43], these results suggest that no or very little damage was generated and accumulated in the joints at the selected load levels. Therefore, one may conclude that the generated peel and peak shear stresses at the edge of the joints were negligible at these load levels. This was in fact due to the formation of the lower bending moment, which is directly proportional to the applied external loads [48] and influences the generation of peel stresses. Furthermore, according to Volkersen [49], low external loads reduced the generated peak shear stresses.

### 3.4. Failure Mode

**Figure 6:**
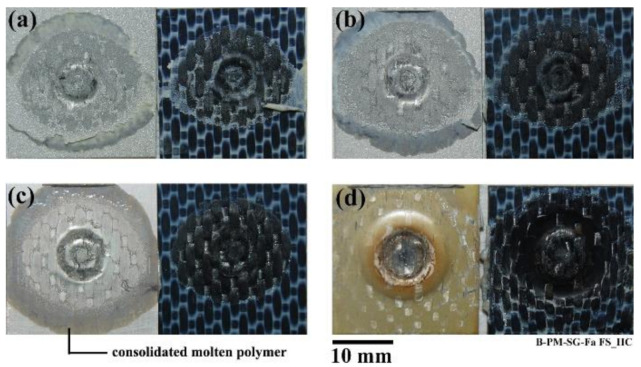
Fracture surface of the HC fatigue specimens: (**a**) SB—606,281 cycles to failure, (**b**) SB + CC—129,406 cycles to failure, (**c**) PAA—482,136 cycles to failure, and (**d**) PAA-P—442,673 cycles to failure.

The primary failure mode in a friction spot joint under quasi-static loading is shear failure through the composite at volumes adjacent to the metal–composite interface [9,19]. Figure 6 illustrates the failure behavior of the joints in the high-cycle fatigue regime for the four pre-treated specimens. The fatigue failure mode is similar to the quasi-static failure of the joints [14]. All the joints failed in shear mode through the composite under all fatigue load levels and for all surface pre-treatments. Radial cracks were initiated in the outer periphery of the joint (consolidated molten polymer) and propagated toward the center of the joint. For the PAA-P specimen (Figure 6d), pieces of the primer layer (yellowish color) were removed and remained attached to the composite, similar to the quasi-static failure [14]. The crack path frequently moved between inside the PPS molten layer and the primer.

**Figure 7:**
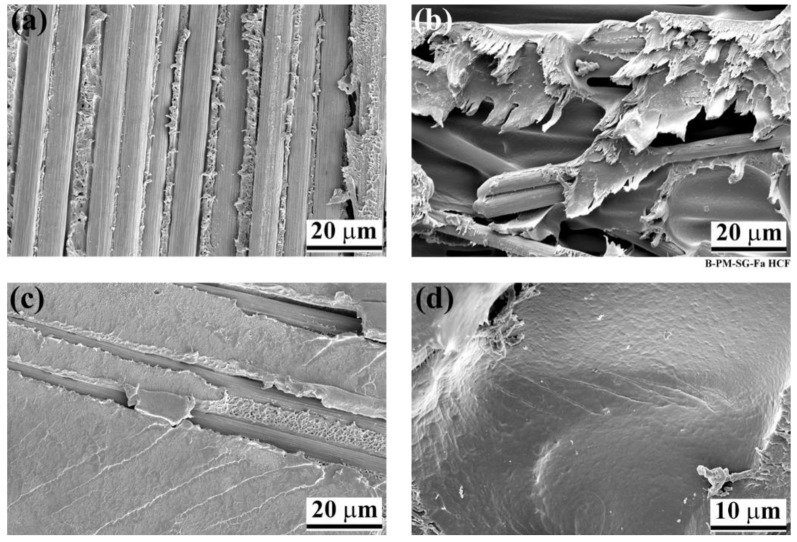
Failure micro-mechanisms of a high-cycle fatigue PAA-P specimen from the composite side: (**a**) warp fibers’ orientation, (**b**) weft fibers’ orientation, (**c**) fatigue striations in the PPS matrix that was detached from the aluminum, and (**d**) striations in the PPS matrix in a resin-rich area (Reprinted from [7] with permission; Copyright Springer Nature 2021). The fatigue loading direction was vertical in all subfigures.

SEM fracture analysis was performed on all specimens. Due to the similarities in failure micro-mechanisms, Figure 7 shows an example of the fracture surface analysis of a PAA-P specimen on the composite. Two types of fracture micro-mechanisms were identified; first, an elongated PPS matrix along the loading path in the warp direction of the composite in between the fibers (Figure 7a), indicating a local ductile fracture; second, fiber–matrix debonding and some fiber breakage along the loading path, but in the weft direction (Figure 7b). Both types of fracture micro-mechanisms were similar to those observed under quasi-static loading, as reported previously [19]. Furthermore, Figure 7c illustrates some fatigue striations on the detached PPS matrix from the aluminum in the weft direction at an angle of 45° to the loading direction. The other type of striations was observed in the resin-rich area in the composite, as shown in Figure 7d.

The first type of striations in Figure 7c was similar to those reported for resistance-welded thermoplastic composites by Dube et al. [43]. Such striations were formed as a result of the generation of peel stresses at the edges of the joint [43]. It can be assumed that with FSpJ, local out-of-plane stresses were formed perpendicular to the plane of the aluminum–PPS interface, leading to the formation of fatigue striations. Moreover, the difference in the local stiffness of the carbon fibers and the PPS matrix led to the crack initiation from micro-voids (generated during the process), as illustrated in Figure 7d. A similar effect was observed by von Bestenbostel and Friedrich [51] on the resin pockets, which are typical defects in the composites. Such resin pockets with different local stiffness than the rest of the composite matrix act as stress concentration and crack initiation sources during the fatigue testing of composites [51].

## 4. Conclusions

This work investigated the fatigue life evaluation of single-lap shear aluminum-composite friction spot joints while considering various aluminum surface pre-treatments. Three models were selected to obtain the S–N curves of the joints. For all surface pre-treatments, all the models fitted well in the experimental range. The exponential model showed to be very conservative in the HCF range, whereas the power-law and wear-out models followed the trend of the experimental data. In the LCF regime, the wear-out model gave the best fit, although it was slightly conservative for the SB, SB + CC, and PAA-P specimens. The trends of the S–N curves for the PAA specimens were similar to the other surface pre-treatments. However, the models showed a more conservative approach, especially for the wear-out model, where the predicted strength was much lower than the quasi-static strength.

The formation of peel stresses at the edges of the joints had an important effect on the fatigue performance of the specimens. It is believed that such peel stresses were much higher for PAA-P specimens compared to the rest of the surface pre-treated samples because of the higher external applied load. Moreover, the presence of sharp-edged asperities as a result of the SB may have increased the local stress concentration at the interface, leading to a reduction in fatigue performance, especially in the high-cycle fatigue regime.

The joints that survived one million cycles did not show any considerable reduction in their quasi-static strength. This was an indication that damage was not accumulated in the joints after one million cycles at the respective load level. Therefore, such a load level (25–35% of the quasi-static strength) can be used as the fatigue endurance limit.

Finally, two types of fatigue striations were observed using SEM. First, striations in the PPS appeared perpendicular to the plane of the aluminum–PPS interface, suggesting the generation of peel stresses at the edges of the joints. Second, striations because of the fatigue crack propagation initiated from micro-voids.

## Figures and Tables

**Table 1:** Fatigue strength (N) of various specimens based on the three models at 10^5^ cycles.

Surface Pre-Treatment	Exponential	Power Law	Wear-Out
SB	846	851	838
SB + CC	1381	1387	1445
PAA	1003	1000	990
PAA-P	2644	2589	2730

## Data Availability

The data presented in this study are available on request from the corresponding author.

## Appendix A. Detailed Fatigue Data from the S–N Curves

Table A1 presents the estimated parameters for the selected fatigue models that were used in this manuscript.

**Table A1:** Estimated parameters for the selected fatigue models.

Surface Pre-Treatment	Exponential (Equation (1))		Power Law (Equation (2))		Wear-Out (Equation (4))			
	a	b	c	d	α	𝛽	C	S
SB	7.16669	−0.00256	25.25512	−6.91255	17.21220	4615.33	0.43191	0.15775
SB + CC	7.90061	−0.00210	31.81210	−8.53354	14.36530	4587.83	0.90861	0.09893
PAA	8.83116	−0.00382	37.1902	−10.72974	6.85346	4210.72	0.65316	0.12544
PAA-P	6.92999	−0.00073	24.9976	−5.85888	7.40972	15653	0.70951	0.15185

## Appendix B. Validation of the Fatigue Models

To validate the models, it was proposed to evaluate the linearity of the exponential and power-law models [29], as well as the goodness-of-fit of the wear-out model [30].

A linearity index was calculated for each of the selected surface pre-treatments and the results are listed in Table A2. These values were compared to the critical linearity index (Fp) taken from [29] for a significance level of 5%. The linearity hypothesis is valid in cases where the linearity index is lower than the Fp. In all cases, the linearity was valid, as can be seen in the table, except for the exponential model with the PAA specimens in which the linearity index exceeded the F_p_. The run-outs were not included in the linearity analysis in accordance with the ASTM standard. It may well be that a slightly higher scattering of the fatigue data in the HCF region (which had a greater impact on the analysis) led to a rejection of the linearity hypothesis. Such behavior was also observed in [27].

**Table A2:** Calculation of the linearity index for the exponential and power-law models.

Surface Pre-Treatment	Exponential		Power Law	
	Linearity Index	Fp	Linearity Index	Fp
SB	4.46024	5.1174	0.59666	5.1174
SB + CC	3.18467	4.7571	3.33068	4.7571
PAA	6.31900	4.7374	2.89993	4.7374
PAA-P	0.11993	4.4590	0.03010	4.4590

An evaluation of the fitting procedure for the wear-out model was performed using an analysis of the goodness-of-fit, as described in [30]. Kruskal–Wallis statistics were performed on the ESS data obtained. Using a Kruskal–Wallis analysis, an H value (H_KW_) was calculated and compared to a critical H value (H_cr_) that corresponded to the 5% significance level. The values of H_cr_ were obtained from [52]. Table A3 lists the calculated H_KW_ and obtained H_cr_. The degree of freedom (DF) in the table corresponds to the number of selected load levels for each surface pre-treatment. The H_KW_ was lower than the H_cr_ for all surface pre-treatments, implying that the fitting procedure of the wear-out model was appropriate. However, for the PAA specimens, H_KW_ was very close to the H_cr_, which could indicate a lower fitting, as was also observed in Figure 3.

**Table A3:** Calculated Kruskal–Wallis statistics index and the respective critical value for the wear-out model.

Surface Pre-Treatment	DF	H_KW_	H_cr_
SB	3	5.47	7.81
SB + CC	5	10.28	11.07
PAA	4	9.26	9.49
PAA-P	4	2.16	9.49
